# Comparing the Diagnostic Efficacy of Different Calcitonin Stimulation Tests for Sporadic Medullary Thyroid Carcinoma: Calcium Gluconate vs. Calcium Chloride

**DOI:** 10.3390/diagnostics15151850

**Published:** 2025-07-23

**Authors:** Jovan Ilic, Katarina Tausanovic, Goran Zoric, Milan Jovanovic, Matija Buzejic, Sara Ivanis, Milan Parezanovic, Milan Marinkovic, Nemanja Karamarkovic, Ana Petakov, Vladan Zivaljevic

**Affiliations:** 1 Clinic for Endocrine Surgery, University Clinical Center of Serbia, 11000 Belgrade, Serbia; jovanilic95@gmail.com (J.I.); katarinatausanovic@gmail.com (K.T.); goranvanjazoric@gmail.com (G.Z.); milanjovanovicceh@gmail.com (M.J.); matijabuzejic@gmail.com (M.B.); saraivaniss@gmail.com (S.I.); milan.parezanovic32@yahoo.com (M.P.); marinkovic.milan1995@gmail.com (M.M.); 2 School of Medicine, University of Belgrade, 11000 Belgrade, Serbia; nemanja_bg89@hotmail.com; 3 Clinic for Cardiosurgery, University Clinical Center of Serbia, 11000 Belgrade, Serbia; 4 Clinic for Endocrinology, Diabetes and Metabolic Diseases, University Clinical Center of Serbia, 11000 Belgrade, Serbia; anapetakov@gmail.com

**Keywords:** medullary thyroid carcinoma, calcitonin, biomarker, stimulation test, calcium gluconate, calcium chloride, endocrine surgery

## Abstract

**Background:** Medullary thyroid carcinoma (MTC) is a rare malignancy derived from parafollicular C-cells, with calcitonin (Ct) as its key biomarker. While basal Ct (bCt) levels above 100 pg/mL strongly suggest MTC, intermediate elevations (10–100 pg/mL) may reflect C-cell hyperplasia (CCH) or other benign conditions, making diagnostics challenging. Although calcium stimulation testing enhances sensitivity, the optimal cut-off values and comparative efficacy of calcium gluconate (CG) versus calcium chloride (CC) remain insufficiently researched. **Methods:** Data on 176 patients who underwent total thyroidectomy between 2009 and 2025 were retrospectively analyzed. BCt values ranged from 10 to 100 pg/mL, and stimulated Ct (sCt) values were above 100 pg/mL. CG was used from 2009 to 2019, and CC was used from 2020 to 2025. Definitive pathohistological findings divided patients into those with MTC, CCH, or no C-cell pathology. Receiver operating characteristic (ROC) analysis identified optimal Ct thresholds for predicting MTC for each stimulatory agent. **Results:** Of the 176 patients, 36 (20.5%) had confirmed MTC. A bCt threshold of 31.1 pg/mL yielded 69.4% sensitivity and 87.1% specificity. For sCt, optimal cut-offs were 810.8 pg/mL for CG and 1076 pg/mL for CC. Lower thresholds (388.4 pg/mL for CG and 431.5 pg/mL for CC) improved sensitivity (≥76.9%) and negative predictive value (>91%). **Conclusions:** Calcium stimulation testing improves MTC detection in patients with moderate bCt elevation. Although CG showed marginally better diagnostic performance, CC remains a practical and reliable alternative, especially when higher cut-off values are considered. Early surgical intervention should be considered when sensitivity-driven thresholds are met.

## 1. Introduction

Medullary thyroid carcinoma (MTC) is a neuroendocrine malignant tumor derived from calcitonin (Ct)-producing C-cells of the thyroid gland [1,2,3]. About 20–25% of MTCs are familial, including multiple endocrine neoplasia (MEN) 2 and its subtypes [3]. The 10-year overall survival rate for patients with MTC ranges from 100% to 21% depending on the stage of the disease (I-IV). If the patient becomes biochemically disease-free (with normalized calcitonin levels), which occurs in 43% of operated cases, the survival rate is excellent, amounting to 97.7% at 10 years [2,4]. The most important biomarker for diagnosing MTC is the measurement of Ct. Normal basal Ct (bCt) levels are typically below 10 ng/mL, with physiological values generally higher in males, likely due to the greater C-cell mass in men [5,6]. The Ct value, which confirms the diagnosis of MTC with nearly 100% specificity, is greater than 100 pg/mL [5,7,8,9]. Ct can be slightly elevated in smokers, in patients taking proton pump inhibitors, and in patients with chronic renal failure, autoimmune thyroiditis, and small-cell lung cancer [10,11,12]. However, the most prevalent entity accountable for a moderate elevation of bCt (10–100 pg/mL) is C-cell hyperplasia (CCH), defined as an increase in the number of C-cells [13]. The two subtypes of CCH are reactive (RCCH) and neoplastic (NCCH) [14], which can be histologically distinguished by the presence of spindle-shaped cells and nuclear polymorphisms present in NCCH [15]. RCCH has no malignant potential [14], but it is seldom present in thyroid tissue with follicular cell malignancies [16]. NCCH, on the other hand, is considered a preneoplastic condition associated with familial forms of MTC [14], although cases of NCCH in patients with no mutation in the RET oncogene have been described [17]. This makes it even more challenging to select cases for thyroidectomy, since every CCH would have the potential to be associated with some thyroid malignancy. In these cases, the stimulation test is performed to increase the sensitivity of Ct testing, either by pentagastrin (Pg), an agent that is no longer available in the US, Europe, and South America [18,19], or by application of intravenous calcium, a more available, cheaper, safer, and potentially more potent secretagogue [19,20,21,22]. However, since there are no widely accepted cut-off values for stimulated Ct (sCt), an overlap between CCH and MTC still occurs [23]. Calcium gluconate (CG) is the most commonly referenced calcium agent in the contemporary literature [6,24]. Although calcium chloride (CC) has previously been described as a secretagogue, it has not been commonly used in recent studies [25,26]. Furthermore, since no published studies have directly compared the diagnostic efficacy of stimulation tests using these two calcium agents, the primary aim of the present study was to evaluate and compare the effectiveness of calcium gluconate and calcium chloride in eliciting a diagnostic biochemical response during stimulation testing.

## 2. Materials and Methods

This retrospective study analyzed clinical data on patients who underwent surgical treatment for elevated calcitonin levels and suspected MTC between January 2009 and January 2025 at the Clinic for Endocrine Surgery, University Clinical Center of Serbia. The data were obtained by reviewing medical histories, including operative and pathohistological (PH) findings using the prospectively maintained electronic database implemented into the institution’s routine clinical practice.

All patients whose bCt was higher than 100 pg/mL underwent surgery. Patients whose bCt levels were between 10 and 100 pg/mL were referred for a calcium stimulation test. In all patients included in the study, other causes of hypercalcitoninemia (use of proton pump inhibitors, renal insufficiency, other neuroendocrine malignancies, pseudohypoparathyroidism, and hypergastrinemia) were excluded. Patients with advanced kidney disease, hypercalcemia, arrhythmogenic cardiac conditions, or a history of myocardial infarction were excluded from undergoing the test due to contraindications. Additionally, patients with a family history of MTC, as well as those with proven MEN 2 syndrome, were excluded from the study.

Patients diagnosed between 2009 and 2019 were tested using CG, while those diagnosed from 2020 to 2025 underwent testing with CC. This series includes data from our previously published study involving 74 patients tested exclusively with CG [27]. Prior to testing, all patients provided informed consent after being informed of the potential side effects. A peripheral venous cannula was placed, and a bCt sample was collected at time 0. The stimulation test was conducted by intravenously administering either 8.5% CG (2.5 mg/kg) or 3% CC (calculated as body mass × 2/8.08) over a 60-second period. Following the injection, blood samples were collected at 1, 3, 5, 8, and 10 min to measure calcitonin levels, with the peak value used for analysis.

According to the definitive PH findings, the operated patients were divided into three groups: those with MTC, CCH, or without any C-cell pathology. Associated thyroid gland pathology was also recorded.

### Statistical Analysis

Statistical analyses were performed using IBM SPSS for Windows, version 25.0. Statistical significance is defined as *p* ≤ 0.05. Numerical results are presented as mean values with standard deviation. An independent samples t-test was used for comparison of numerical values among different groups. Pearson’s correlation coefficient with bootstrapping was performed for correlation testing, and the scatterplot was used for graphical presentation of the data.

Optimal Ct cut-off values that can reliably diagnose MTC preoperatively were determined using receiver operator characteristics (ROC) analysis and calculating the Youden index (= sensitivity + specificity − 1) for three diagnostic groups: (1) bCt, (2) sCt after the application of CG, and (3) sCt after the application of CC. The strength of the statistic test was determined by the area under the curve (AUC). Sensitivity (Sn) and specificity (Sp) were calculated and presented as percentages, with specificity defined as 1—sensitivity. The positive predictive value (PPV) and negative predictive value (NPV) were subsequently determined using the optimal Ct cut-off values based on the following formulas: PPV = true positives/(true positives + false positives); NPV = true negatives/(true negatives + false negatives).

## 3. Results

Between January 2009 and January 2025, a total of 12,284 patients were operated on in our clinic for various thyroid gland diseases. During this period, MTC was confirmed in 272 patients, making the prevalence of MTC in our series 2.2%.

A total of 176 patients (106 women and 70 men—Table 1), in whom bCt levels were between 10 and 100 pg/mL and in whom Ct spikes were over 100 pg/mL on the calcium stimulation test, were included in the study. The participants were between 23 and 79 years old, with an average age of 56 years. There were 123 individuals aged 50 and older and 53 individuals younger than 50 (Table 1). All patients had morphological changes on ultrasound in the thyroid gland. In total, 66 patients (37.5%) had a solitary nodule, 52 patients (29.5%) had micronodal changes in both thyroid lobes, 34 patients (19.3%) had a multinodular goiter, and 24 patients (13.6%) had diffuse goiter, usually due to underlying autoimmune disease (Graves’ disease or Hashimoto thyroiditis—HT). The size of the nodules, as measured by ultrasound examination, ranged from 3.3 to 44.0 mm, with an average of 14.2 ± 7.7 mm. All patients underwent a preoperative ENT examination. Twenty-three patients had overt hypothyroidism and preoperatively received l-thyroxine replacement therapy in doses from 25 to 150 mcg. Another four patients had a subclinical form of HT.

All patients included in the study underwent total thyroidectomy. Among them, 13 patients (7.6%) underwent unilateral central neck dissection, while 10 patients (5.8%) underwent bilateral central neck dissection. In our series, none of the patients underwent lateral neck dissection.

The average bCt level was 25.3 ± 14.6 pg/mL (10.0 to 81.2 pg/mL). The average peak Ct level after stimulation was 667.1 ± 462.4 pg/mL (108.0 to 3007.0 pg/mL). There was no statistically significant difference between the bCt and sCt values across genders. However, a statistically significant difference in Ct values was observed before and after stimulation with calcium agents, especially when comparing patients younger than 50 years old to those aged 50 years or older (Table 1).

### 3.1. Confirmed Cases of MTC

Based on postoperative PH analysis, 36 patients (20.5%) had MTC, while in 119 patients, CCH was found. Twenty-one patients had neither MTC nor CCH but other benign or malignant thyroid disorders, such as papillary thyroid carcinoma, follicular adenoma, thyroid follicular nodular disease, HT, or follicular adenoma (Table 2). The characteristics of patients with a verified diagnosis of MTC are summarized in Table 3.

The patients were divided into three groups: confirmed MTC, CCH, and those without C-cell pathology (Table 4). In patients with MTC, the mean bCt value was 38.6 ± 18.8 pg/mL (12.9 to 81.2 pg/mL), while the Ct value after stimulation with either CG or CC was 1029.8 ± 684.44 pg/mL (185.0 to 3007.0 pg/mL). In patients with PH-confirmed CCH, the bCt value was 21.8 ± 9.8 pg/mL (10 to 65.2 pg/mL), while the Ct value after stimulation with CG or CC was 581.7 ± 316.6 pg/mL (from 108.0 to 1818.0 pg/mL). In patients with other thyroid disorders, the bCt was 22.1 ± 17.6 pg/mL (11.0 to 75.8 pg/mL), while the mean Ct value after stimulation with CG or CC was 525.5 ± 371.0 pg/mL (122.0 to 1654.0 pg/mL) (Table 4).

**Figure 1:**
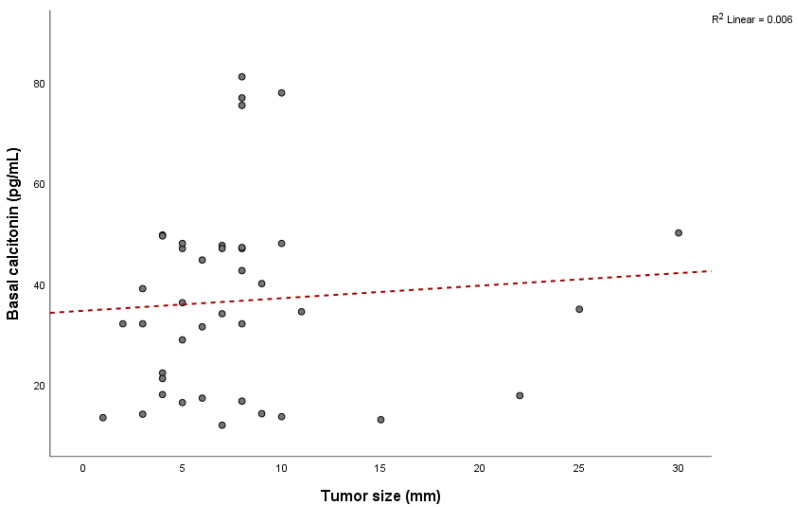
Relationship between tumor size and basal calcitonin values.

**Figure 2:**
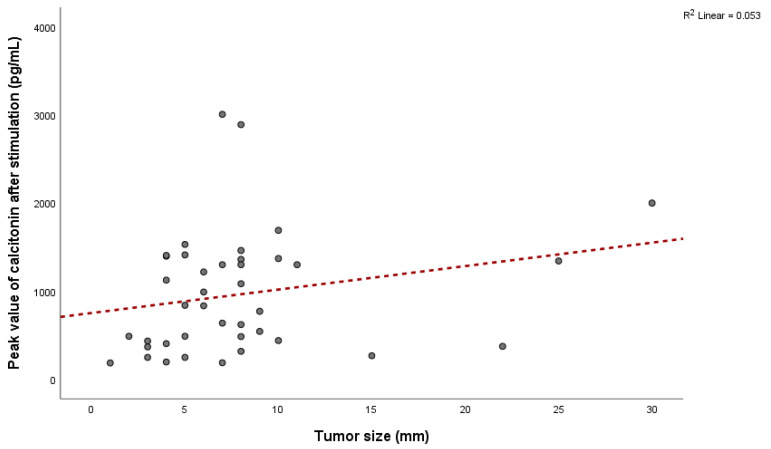
Relationship between tumor size and peak values of stimulated calcitonin.

Among patients with MTC, 31 (86.1%) were female and 5 (13.9%) were male, with a mean age of 59.2 ± 10.8 years (range: 36 to 78 years), indicating that 29 patients (80.6%) were older than 50. Mean values of bCt in female patients with confirmed MTC were 40.7 ± 18.7 pg/mL, while it was 25.5 ± 14.8 pg/mL in male patients. The mean value of sCt in females was 1069.5 ± 684.1 pg/mL, and it was 781.1 ± 706.5 pg/mL in males. There were no significant differences between genders for bCt (*p* = 0.09) and sCt (*p* = 0.39). The average tumor size in patients with confirmed MTC was 7.7 ± 5.6 mm (ranging from 1 to 30 mm) and did not correlate with bCt levels (Pearson = 0.08, *p* = 0.64) (Figure 1) or sCt levels (Pearson = 0.23, *p* = 0.15) (Figure 2).

### 3.2. ROC Analysis

ROC analysis was performed to identify a Ct threshold capable of distinguishing patients with MTC from other patients (patients with CCH and without C-cell pathology).

**Figure 3:**
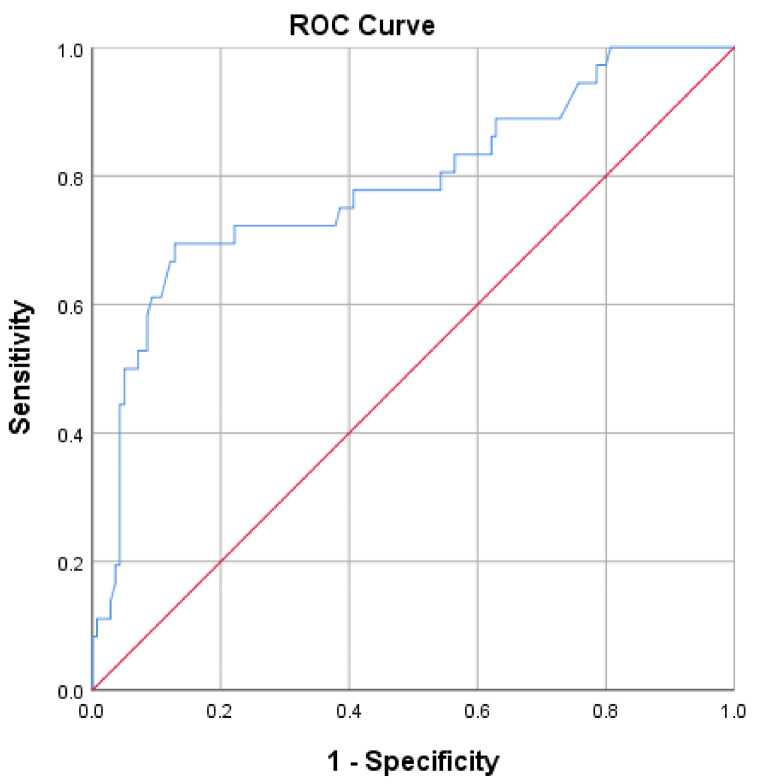
ROC plot analysis of basal calcitonin.

A bCt value of 31.1 pg/mL effectively distinguishes patients with MTC from others with an Sn of 69.4% and an Sp of 87.1% (*p* < 0.001; AUC = 0.786; 95%CI = 0.69–0.88; Youden index = 0.57) (Figure 3). The PPV for this cut-off value of bCt is 55%, and the NPV is 91.6%. Should sensitivity be raised, a lower calcitonin value of 16.2 pg/mL could serve as a cut-off (Sn = 88.9%; Sp = 37.1%; PPV 26.7%; NPV 92.9%) (Table 5).

**Figure 4:**
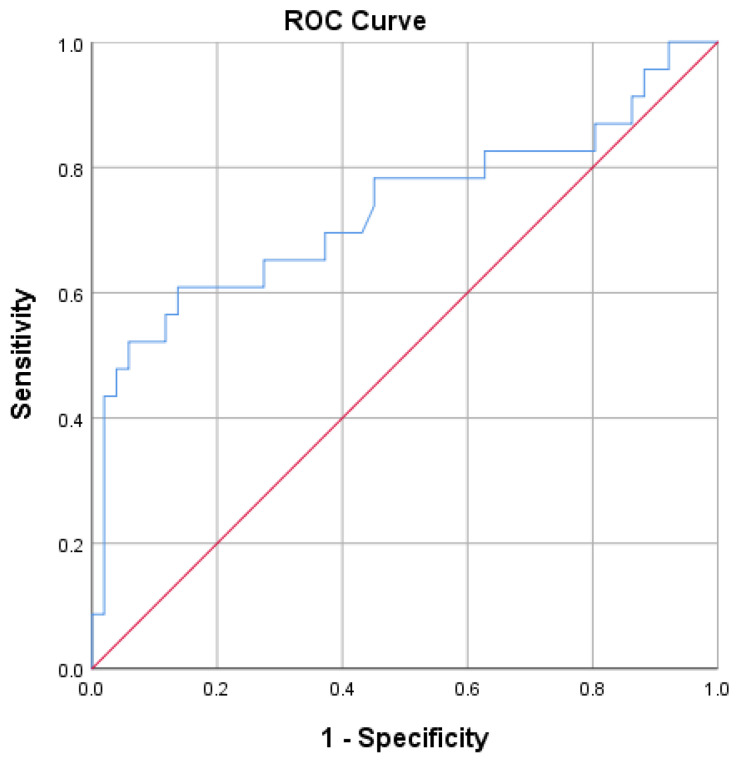
ROC plot analysis of stimulated calcitonin after application of Ca-gluconate.

An sCt value of 810.8 pg/mL after Ca-gluconate stimulation can help differentiate between patients with MTC and those with other thyroid diseases with an Sn of 60.9% and an Sp of 87.3% (*p* = 0.001; AUC = 0.733; 95%CI = 0.59–0.87; Youden index = 0.47) (Figure 4). Using this cut-off value, the PPV for sCt testing with CG is 66.7% and the NPV is 79.2%. If a higher sensitivity is desired, a lower sCt value of 388.4 pg/mL could be considered as an alternative cut-off, with a sensitivity of 82.6%, a specificity of 37.3%, a PPV of 37.3%, and an NPV of 82.6% (Table 6).

**Figure 5:**
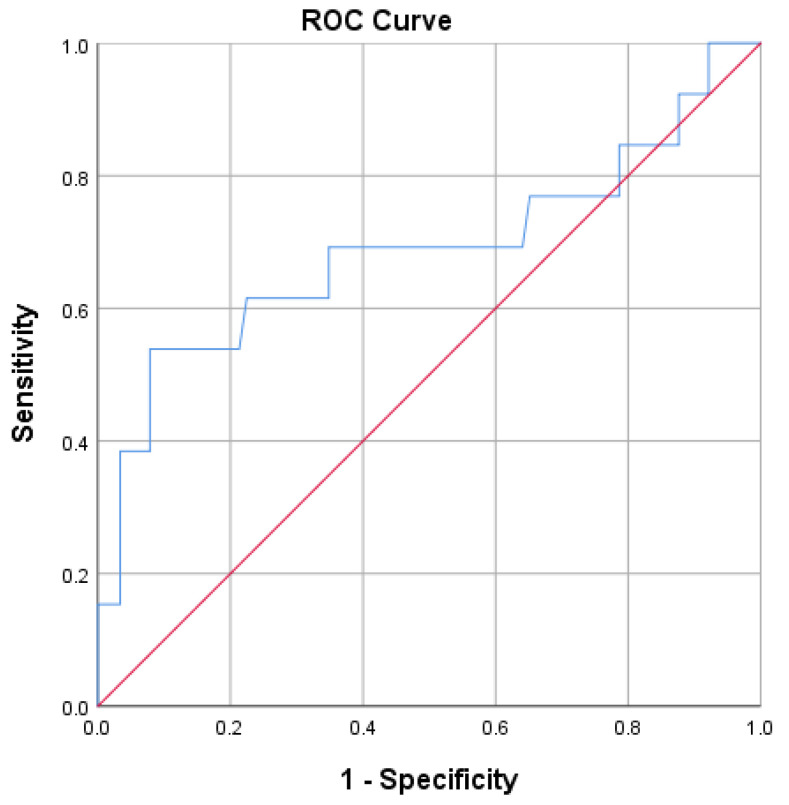
ROC plot analysis of stimulated calcitonin after application of Ca-chloride.

An sCt value of 1076 pg/mL after Ca-chloride stimulation makes it possible to distinguish between patients with MTC and those with other thyroid diseases with an Sn of 53.8% and an Sp of 92.1% (*p* < 0.05; AUC = 0.688; 95%CI = 0.49–0.88; Youden index = 0.46) (Figure 5). Using this cut-off value, the PPV for sCt testing with Ca-chloride is 50% and the NPV is 93.2%. With the Sn increasing to 76.9% and an Sp of 34.8%, the cut-off value is 431.5 pg/mL (PPV 14.7%; NPV 91.2%) (Table 7).

## 4. Discussion

The present study was designed and conducted to investigate whether the use of different calcium secretagogues in stimulating tests influences Ct cut-off values and to what extent. The study aimed to investigate the diagnostic strength of the stimulating tests using CC compared to CG.

Although rare, MTC represents an important clinical entity due to its unique biological behavior, histopathological characteristics, and frequent hereditary presentation. Those facts highlight the importance of obtaining a timely diagnosis. In our series, the prevalence for MTC was 2.2%. Reported prevalence rates typically range from 3% to 10% of all thyroid cancers [2,28]. However, with advances in the diagnosis and treatment of papillary thyroid carcinoma, the relative prevalence of MTC has recently declined to 1–2%, which is consistent with our findings [8,29,30].

Most patients with sporadic forms of MTC are diagnosed between the fourth and fifth decade of life, and there is slight female predominance (1:1.3) [5,31]. In our study, female patients outnumbered male patients by a ratio of six to one, with over 80% being older than 50 years. Mean values of bCt and sCt in female patients were higher than in males, with values of 40.7 ± 18.7 pg/mL vs. 25.5 ± 14.8 pg/mL and 1069.5 ± 684.1 pg/mL vs. 781.1 ± 706.5 pg/mL, respectively. The bCt and sCt values did not differ significantly between genders within our cohort (*p* = 0.09 for bCt; *p* = 0.39 for sCt). This result is inconsistent with the existing literature data, which predominantly reports statistically significant differences between genders, namely higher values in males, which affects cut-off values of Ct in daily clinical practice. The advocates of using separate cut-off values for males and females in stimulation tests had a more acceptable ratio of males to females [24,32], while in our study, the demographic data is based on only 36 cases with moderate calcitonin elevation, representing just 13.2% of all confirmed MTC cases in our clinic, with a significant dominance of female patients (86.1%), which influenced the mean values, and restricts the use of gender-specific Ct cut-offs.

In our study, nine patients with confirmed MTC (25%) had either a subclinical or an overt form of HT. Zayed et al. suggest that there is no higher incidence of MTC in patients with HT in the general population. However, a statistically significant association was found in female patients, although this finding was based on a limited number of cases [33]. Machens et al. disproved the association between CCH and HT in a study conducted on patients operated on at their center [34]. Shuetz et al. state that the prevalence of both MTC and CCH in patients with HT is 0.35%, which is lower than in patients with nodular disease [11]. Most of the evidence on the association between MTC and CCH and HT is reported in sporadic case reports [35,36,37,38,39,40].

Besides its local invasiveness and potency to lymph node and distant metastases, one characteristic that makes MTC remission difficult to achieve is the lack of radioiodine avidity [41], which is why a certain dose of radioiodine is recommended on initial surgical treatment. Since our study included only patients with moderately elevated bCt levels (10–100 pg/mL), and without ultrasonographic findings of lateral lymph node metastases (LNM), all patients had a total thyroidectomy performed, with 13 patients (7.6%) undergoing unilateral central dissection and 10 patients (5.8%) undergoing bilateral central dissection. No functional dissection of lateral lymph node metastasis (LNM) was performed, which is to be expected, as patients with higher bCt levels (>100 pg/mL) are typically those in whom lateral neck LNM is found. Park et al., in a large-scale study assessing the predictive value of basal calcitonin (bCt) levels for lymph node metastases (LNM), found no cases of lateral LNM and only five cases of central LNM in patients with bCt levels below 100 pg/mL. The group of authors also concluded that preoperative bCt levels can help guide the extent of initial surgical management [42]. Bae et al. propose Ct values of 226 pg/mL as a cut-off for ipsilateral central LNM, and 237 pg/mL for ipsilateral lateral LNM, with even higher values for contralateral LNM [43].

Prinzi et al. found a positive correlation between Ct levels and the T stage of tumors, with a Ct value of 60 pg/mL being the median for the T1a stage [44]. Numerous other studies have strongly supported this correlation [42,45,46,47,48]. In our study, 32 out of 36 confirmed MTC cases measured 1 cm or less, with a mean tumor size of 7.7 ± 5.6 mm and a mean basal calcitonin (bCt) level of 38.6 ± 18.8 pg/mL. The correlation between a Ct value and the size of medullary carcinoma was not found. Based on the highest calculated Youden index, we identified a bCt level of 31.1 pg/mL as the optimal cut-off for diagnosing MTC in this patient group, with a PPV of 55%. The values of 810.8 pg/mL (66.7% PPV) and 1076 pg/mL (50% PPV) were optimal thresholds for sCt, using CG and CC, respectively (Figure 3, Figure 4 and Figure 5; Table 5, Table 6 and Table 7).

Table 8 presents a comparison of our findings with those of similar studies that propose bCt and sCt cut-off values following CG stimulation testing.

A review of the literature on stimulatory tests performed with CC indicates that this method has been abandoned, since the studies in which CC is used (either on its own or in combination with Pg) span from 1974 to 1984. Even then, the studies were often conducted on healthy subjects who had no confirmed MTC, with the main focus of Ct research being due to its believed role as a calcemic peptide hormone [25,26,51].

Among the limited and dated data, McLean et al. proposed a stimulated calcitonin (sCt) cut-off value of 800 pg/mL for patients without a family history of MTC [52]. Wells et al. found CC stimulation to be less effective, but only relative to combined CG and Pg stimulation [53]. VanLathem et al. compared sCt levels in asymptomatic relatives of confirmed MEN2A patients to those in a control group of healthy individuals without a family history of MTC, using CC in combination with Pg [54]. However, their findings do not apply to our discussion, as our study included only cases of sporadic MTC, and none of the patients had a healthy thyroid gland. This positions our study as the first to directly compare stimulating tests using two different agents, both containing calcium as the active component, in a patient population for whom such testing is most relevant—those with moderately elevated basal calcitonin levels (10–100 pg/mL) and sporadic MTC.

The CC stimulation test was slightly inferior compared to the CG stimulation test (AUC 0.69 vs. 0.73), with slightly higher cut-off values needed to diagnose MTC-1076 vs. 810.8 pg/mL if a lower Sn value is obtained, or 431.5 vs. 388.4 pg/mL with a higher Sn value. This makes testing with CG somewhat more advantageous.

This study has several limitations that should be acknowledged. First, the analysis did not account for gender-specific calcitonin cut-off values, despite those being addressed in several previously cited studies. This decision was based on the absence of statistically significant differences in bCt and sCt levels between genders, the marked female predominance in our cohort (Table 1), and the primary objective of the study—to compare the diagnostic performance of two different stimulatory agents. Second, the study population was limited to patients with moderately elevated basal calcitonin levels (10–100 pg/mL), which may have contributed to the slightly lower AUC values observed when compared to studies with broader inclusion criteria (Table 8). Third, due to the retrospective design of the study and the non-randomized assignment of the secretagogue, the same patients were not tested with both CG and CC, which prevented a direct, within-subject comparison of the two stimulation tests. A prospective study, carried out on a larger patient pool, matched by gender, in which the same patients undergo both stimulation tests, spaced one week apart, would address these limitations, enable the use of gender-specific cut-off values, and prevent any possibility of bias. Our research group may consider such a design in the future, which may be contingent on the availability of CG.

## 5. Conclusions

Given the therapeutic challenges and overall aggressiveness of MTC, we recommend using cut-off values with higher sensitivity to guide initial surgical decisions in patients with moderately elevated basal calcitonin levels. These values are 16.2 pg/mL for bCt, 388.4 pg/mL for sCt following CG stimulation, and 431.5 pg/mL for sCt following CC stimulation. Although the CC stimulation test demonstrated slightly lower diagnostic performance compared to the CG test, it can still be reliably used in clinical practice, with awareness of its marginally higher cut-off threshold.

## Figures and Tables

**Table 1:** Comparison of basal and stimulated calcitonin values based on patient demographics.

	Sex			Age (Years)		
Demographic	Male	Female	*p*-value	<50	≥50	*p*-value
N	70	106		53	123	
Basal Ct	25.2 ± 11.6	25.3 ± 16.4	0.97	20.6 ± 12	27.2 ± 15.2	<0.01
Stimulated Ct	669.5 ± 396.9	663.4 ± 497.5	0.9	534.3 ± 330.9	724.3 ± 499.1	0.01

**Table 2:** Distribution of patients who were operated on based on pathohistological diagnosis.

Pathohistological Diagnosis	Number of Patients	%
Medullary thyroid carcinoma	36	20.5
C-cell hyperplasia	119	67.6
Papillary thyroid carcinoma	5	2.8
Thyroid follicular nodular disease	10	5.7
Hashimoto thyroiditis	4	2.3
Follicular adenoma	2	1.1

**Table 3:** Characteristics of patients with a verified diagnosis of MTC.

No.	Sex	Age (Years)	TNM	Tu Size (mm)	Test	Basal Ct (pg/mL)	Stimulated Ct (pg/mL)	Associated Pathology
1	Female	59	T1NxMx	4	Ca-gluconate	22.2	195.8	HT
2	Female	68	T1NxMx	5	Ca-gluconate	16.3	248.0	TFNB
3	Male	63	T2NxMx	15	Ca-gluconate	12.9	266.6	TFNB
4	Female	61	T1NxMx	8	Ca-gluconate	42.6	317.0	Follicular adenoma
5	Female	66	T1NxMx	4	Ca-gluconate	17.9	403.7	TFNB
6	Female	63	T1NxMx	8	Ca-gluconate	16.6	483.9	TFNB
7	Female	55	T1NxMx	5	Ca-gluconate	28.8	488.0	TFNB
8	Female	59	T1NxMx	9	Ca-gluconate	14.1	542.8	TFNB
9	Female	72	T1NxMx	8	Ca-gluconate	81.2	620.4	HT
10	Female	71	T1NxMx	6	Ca-gluconate	31.4	833.5	TFNB
11	Female	54	T1NxMx	5	Ca-gluconate	36.2	839.3	HT
12	Female	66	T1NxMx	6	Ca-gluconate	17.2	990.0	TFNB
13	Female	51	T1NxMx	6	Ca-gluconate	44.7	1217.3	HT
14	Female	66	T1NxMx	7	Ca-gluconate	47.6	1300.0	TFNB
15	Female	76	T2NxMx	25	Ca-gluconate	34.9	1342.1	PTC
16	Female	64	T1NxMx	8	Ca-gluconate	47.0	1360.0	TFNB
17	Female	42	T1NxMx	10	Ca-gluconate	78.0	1370.0	HT
18	Male	45	T1NxMx	4	Ca-gluconate	21.1	1396.9	TFNB
19	Female	60	T1NxMx	5	Ca-gluconate	47.0	1411.0	TFNB
20	Female	72	T1NxMx	8	Ca-gluconate	47.2	1461.4	TFNB
21	Female	61	T1NxMx	5	Ca-gluconate	48.0	1530.0	TFNB
22	Male	56	T1NxMx	10	Ca-gluconate	48.0	1690.0	TFNB
23	Female	63	T1NxMx	7	Ca-gluconate	47.0	3007.0	TFNB
24	Female	44	T1NxMx	4	Ca-chloride	49.7	1125	TFNB
25	Female	46	T1N1Mx	11	Ca-chloride	34.4	1300	HT
26	Female	36	T1NxMx	3	Ca-chloride	14.0	248	HT
27	Female	53	T1NxMx	3	Ca-chloride	39.0	433	TFNB
28	Male	44	T1NxMx	3	Ca-chloride	32.0	367	PTC
29	Female	52	T1NxMx	9	Ca-chloride	40.0	771	HT
30	Female	51	T1NxMx	4	Ca-chloride	49.5	1405	TFNB
31	Female	68	T2NxMx	30	Ca-chloride	50.1	2000	PTC
32	Female	70	T1NxMx	8	Ca-chloride	75.5	184	HT
33	Female	78	T1NxMx	7	Ca-chloride	34.0	637.0	TFNB
34	Female	70	T1NxMx	8	Ca-chloride	77.0	2890	TFNB
35	Female	66	T1NxMx	8	Ca-chloride	32.0	1300	TFNB
36	Male	40	T1NxMx	1	Ca-chloride	34.0	893	PTC

Tu—tumor; Ct—calcitonin; HT—Hashimoto thyroiditis; TFNB—thyroid follicular nodular disease; PTC—papillary thyroid carcinoma.

**Table 4:** Basal and stimulatory calcitonin values in all three groups of patients.

PH Diagnosis	Basal Calcitonin (pg/mL)	Stimulated Calcitonin (pg/mL)
Medullary thyroid carcinoma	38.6 ± 18.8 (12.9–81.2)	1029.8 ± 684.4 (185.0–3007.0)
C-cell hyperplasia	21.8 ± 9.8 (10–65.2)	581.7 ± 316.6 (108.0–1818.0)
Other diseases of the thyroid gland	22.1 ± 17.6 (11.0–75.8)	525.5 ± 371.0 (122.0–1654.0)

**Table 5:** Diagnostic test parameters for basal Ct using different cut-off values.

Cut-Off (pg/mL)	AUC	Sensitivity	Specificity	PPV	NPV
31.1	0.79	69.4%	87.1%	55%	91.6%
16.2		88.9%	37.1%	26.7%	92.9%

**Table 6:** Diagnostic test parameters for Ca-gluconate sCt using different cut-off values.

Cut-Off (pg/mL)	AUC	Sensitivity	Specificity	PPV	NPV
810.8	0.73	60.9%	87.3%	66.7%	79.2%
388.4		82.6%	37.3%	37.3%	82.6%

**Table 7:** Diagnostic test parameters for Ca-chloride sCt using different cut-off values.

Cut-Off (pg/mL)	AUC	Sensitivity	Specificity	PPV	NPV
1076	0.69	53.8%	92.1%	50%	93.2%
431.5		76.9%	34.8%	14.7%	91.2%

**Table 8:** Diagnostic test parameters for basal and Ca-gluconate-stimulated Ct using different cut-off values among different research studies.

Ilic et al. (2025)	AUC	Cut-Off (pg/mL)	Sensitivity	Specificity	PPV	NPV
Basal Ct-H	0.79	31.1	69.4%	87.1%	55%	91.6%
Basal Ct-L		16.2	88.9%	37.1%	26.7%	92.9%
Stimulated Ct-H	0.73	810.8	60.9%	87.3%	66.7%	79.2%
Stimulated Ct-L		388.4	82.6%	37.3%	37.3%	82.6%
Faggiano et al. (2023) [9]						
Basal Ct-F	0.8	19.2	75%	82%	84%	72%
Stimulated Ct-F	0.79	445	54%	100%	100%	63%
Basal Ct-M	0.76	39	67%	97%	86%	91%
Stimulated Ct-M	0.9	611	67%	100%	100%	91%
Fugazzola et al. (2021) [49]						
Basal Ct-F	0.91	30	75.9%	93.7%	88%	86.5%
Stimulated Ct-F	0.84	79	100%	50%	53.8%	100%
Basal Ct-M	0.97	34	88.9%	95%	88.9%	92.6%
Stimulated Ct-M	0.90	466	94.4%	80%	68%	94.2%
Niederle et al. (2020) [50]						
Basal Ct-F	0.94	23	81%	100%	100%	83%
Stimulated Ct-F	0.87	780	69%	100%	100%	76%
Basal Ct-M	0.89	43	53%	100%	100%	67%
Stimulated Ct-M	0.85	1500	55%	100%	100%	68%
Rosario and Calsolari (2017) [18]						
Basal Ct-H	/	47	50%	100%	100%	92.1%
Basal Ct-L	/	24.6	100%	74.3%	40%	100%
Stimulated Ct-H	/	655.2	33.3%	100%	100%	89.7%
Stimulated Ct-L	/	186.5	100%	60%	30%	100%
Mian et al. (2014) [24]						
Basal Ct-F	0.95	26	81.8%	97.9%	94.7%	92%
Stimulated Ct-F	0.93	79	100%	76.6%	68.7%	100%
Basal Ct-M	0.94	68	83.3%	100%	100%	92.9%
Stimulated Ct-M	0.94	544	77.8%	85.4%	68.4%	89.2%
Colombo et al. (2012) [19]						
Basal Ct-F	1	18.7	100%	100%	100%	100%
Stimulated Ct-F	0.98	184	100%	92.9%	6.6%	100%
Basal Ct-M	1	68	100%	100%	100%	100%
Stimulated Ct-M	0.93	1620	75%	100%	100%	99.9%

AUC—area under the curve; PPV—positive predictive value; NPV—negative predictive value; H—higher cut-off value; L—lower cut-off value; F—female; M—male.

## Data Availability

The original contributions presented in this study are included in the article. Further inquiries can be directed to the corresponding author.
